# Acute high-fat feeding leads to disruptions in glucose homeostasis and worsens stroke outcome

**DOI:** 10.1177/0271678X17744718

**Published:** 2017-11-24

**Authors:** Michael J Haley, Siddharth Krishnan, David Burrows, Leon de Hoog, Jamie Thakrar, Ingo Schiessl, Stuart M Allan, Catherine B Lawrence

**Affiliations:** Faculty of Biology, Medicine and Health, The University of Manchester, Oxford Road, Manchester, UK

**Keywords:** Brain ischaemia, high-fat diet, glucose, hyperglycemia, inflammation, lipids, stroke

## Abstract

Chronic consumption of diets high in fat leads to obesity and can negatively affect brain function. Rodents made obese by long-term maintenance on a high-fat diet have worse outcome after experimental stroke. High-fat consumption for only three days does not induce obesity but has rapid effects on the brain including memory impairment. However, the effect of brief periods of high-fat feeding or high-fat consumption in the absence of obesity on stroke is unknown. We therefore tested the effect of an acute period of high-fat feeding (three days) in C57B/6 mice on outcome after middle cerebral artery occlusion (MCAo). In contrast to a chronic high-fat diet (7.5 months), an acute high-fat diet had no effect on body weight, adipose tissue, lipid profile or inflammatory markers (in periphery and the brain). Three days of high-fat feeding impaired glucose tolerance, increased plasma glucose and insulin and brain expression of the glucose transporter GLUT-1. Ischaemic damage was increased (48%) in mice fed an acute high-fat diet, and was associated with a further reduction in GLUT-1 in the ischaemic hemisphere. These data demonstrate that only a brief period of high-fat consumption has a negative effect on glucose homeostasis and worsens outcome after ischaemic stroke.

## Introduction

Chronic consumption of diets high in saturated fat and sugars (Western style diet) can lead to chronic peripheral diseases including obesity that are detrimental to health. High-fat diets can also negatively affect the cerebral microvasculature (e.g. reduce vasodilatory and enhance vasoconstrictive responses), modify neuronal physiology and impair learning and memory in both humans and rodents.^1–4^ Effects of a high-fat diet on memory in rats can occur very quickly (as early as three days) and before weight gain and obesity develop,^5^ suggesting that the diet per se can induce rapid changes in the brain. Obesity is an independent risk factor for stroke, and in rodents, diet-induced obesity leads to greater ischaemic damage.^6–8^ Furthermore, obese rodents often show increases in blood–brain barrier permeability and white matter damage, cerebral oedema and haemorrhagic transformation following experimental stroke.^6,7,9–16^

Obesity in patients is usually due to chronic and excessive consumption of diets high in fat, often resulting in several components of the metabolic syndrome, including hyperglycaemia, hypertension and dyslipidaemia, all of which can affect stroke outcome. Experimental stroke studies reporting worse outcome in obesity typically feed rodents a high-fat diet for two months or greater (e.g. see literature^7,15–20^). Such a long-term high-fat diet results in animals developing some components of the metabolic syndrome, which likely contribute to the worse outcome after experimental stroke. However, it is yet to be determined fully whether the worse stroke outcome observed is due to the obese phenotype that results after chronic high-fat feeding, or due to an acute effect of high-fat diet per se. Experimental and clinical studies show that acute high-fat diets or excessive calorie intake for only three to four days do not cause obesity but can lead to problems in glucose homeostasis including glucose intolerance or insulin resistance,^21–24^ all of which are associated with worse stroke outcome.^25^ Some types of fats, in particular saturated fatty acids, may directly activate inflammatory signalling pathways in metabolically active tissues,^26,27^ and studies suggest that inflammation contributes to impairment in glucose homeostasis after an acute high-fat diet.^23^ Therefore, the aim of this study was to compare the effects of acute (3 days) and chronic (7.5 months) high-fat feeding on components of the metabolic syndrome, glucose homeostasis and inflammation. We have shown previously that high-fat feeding for greater than four months in mice causes obesity and increases ischaemic damage,^7^ so we also determined the effect of an acute high-fat diet (three days) in the absence of obesity on stroke outcome.

## Material and methods

### Animals and diets

C57BL/6 male mice (Harlan UK Limited, UK) at eight weeks of age were randomly assigned a control diet (12% energy from fat, 5% fat content by weight, 0.78% saturated fatty acids, 58G7, Test Diets) or high-fat diet (chronic high-fat; 60% energy from fat, 35% fat content by weight, 13% saturated fatty acids, 58G9, Test Diets) and maintained for 7.5 months. A further group was maintained on a control diet for 7.5 months and then transferred to a high-fat diet for three days (acute high-fat). Mice were then randomly assigned to undergo sham-surgery (*n* = 5–7/diet group), middle cerebral artery occlusion (MCAo, *n* = 13–14/diet group) or no surgical intervention (naïve, *n* = 6–7/diet group). The group of naïve mice was used to measure physiological parameters (blood pressure and glucose tolerance test that were not performed in mice undergoing MCAo or sham surgery), liver and adipose tissue histology and cytokine levels, and blood physiological parameters (see below). Mice were housed on a 12-h light, 12-h dark cycle at a constant ambient temperature of 21 ± 2℃ and given ad libitum access to water and their respective diets. All procedures were conducted in accordance with the United Kingdom Animals (Scientific Procedures) Act, 1968 and approved by the local Animal Welfare and Ethical Review Board, University of Manchester, UK.

### Physiological parameters

After maintenance on respective diets, systolic blood pressure was assessed using a non-invasive blood pressure analyser with a specific mouse tail cuff adapter (Model BP-2000-M-2, Visitech Systems, Inc., USA) in the naïve group of mice (*n* = 6–7/diet group). One day prior to measurements, mice were habituated to the procedure for 5 min to reduce stress and anxiety. On the day of assessment, the animals were placed in the room and allowed to acclimatise for 10 min. A total of 20 readings of systolic blood pressure (mmHg) and heart rate (beats/min, bpm) were taken and the average of the last 10 measurements calculated.

A glucose tolerance test was then performed in mice that had been fasted for 6 h. Glucose concentrations were measured in tail vein samples at 0 min using a hand-held glucose monitor (Accu-Check Aviva, Roche, UK). Mice then received an oral injection of 2 g/kg glucose (Sigma-Aldrich, UK) and blood glucose was measured in tail vein blood after 15, 30, 60 and 120 min and area under the curve calculated.

### Liver and adipose histology

After fixation in 4% PFA, liver and adipose tissue were transferred to 70% ethanol before embedding in paraffin wax. Tissue was cut into 5 µm sections on a rotary microtome (#RM23225, Leica, Germany), mounted onto slides and allowed to dry. Paraffin was removed using xylene and the sections were hydrated using a series of decreasing ethanol concentrations (100%, 90% and 70%). Sections were then stained with Gill3 Haematoxylin (ThermoShandon, Runcorn, UK) for 2 min and staining enhanced by differentiating in acid alcohol (1% hydrochloric acid in 70% ethanol) and bluing in Scott’s Tap Water (2% MgSO_4_, 0.25% NaHCO_3_). Slides were rinsed in running water between each step then cleaned in 70% ethanol. Sections were then stained using Eosin Y (ThermoShandon) (33.5% stock, 1.3% Acetic Acid in 80% ethanol) for 30 s before dehydration in ascending concentrations of ethanol (70%, 90% and 100%), cleaning in xylene and coverslipped using DPX mounting medium. Liver sections were assessed for liver steatosis using criteria previously established (<5%, grade 0, 5–33%, grade 1, 33–66%, grade 2 and >66%, grade 3).^28^

For adipose tissue, images (three random fields of view) were collected from each animal and analysed using image J. The area of each adipocyte (µm^2^) across the three fields of view was calculated using Adiposoft automated analysis software^29^ on the Fiji ImageJ platform. The number of crown-like structures,^30^ which are indicative of macrophages clusters around dead adipocytes, was also counted. Any group of six cells with round nuclei surrounding the adipocyte in a cluster was deemed to be a crown-like structure. One naïve chronic high-fat fed mouse had damaged adipose tissue due to sample preparation, so for these analyses *n* = 5 for this group. All liver and adipose sections were assessed blinded to the experimental groups (diet).

### Blood physiological analysis

Plasma levels of insulin (Crystal Chem Inc., USA), free fatty acids (FFAs; Zen-Bio Inc., USA), glycerol (Sigma-Aldrich, USA) and triglycerides (BioVision Inc., USA) were measured using the relative assays according to the manufacturer’s instructions.

### Middle cerebral artery occlusion

Transient MCAo was used to induce focal ischaemia. In brief, mice were placed under anaesthesia using isoflurane (3% in a mixture of 30% O_2_ and 70% N_2_O) and the carotid arteries were exposed. A 6–0 silicon rubber-coated monofilament (Doccol, USA) with a 4.5 mm tip was inserted into the external carotid artery and advanced along the internal carotid artery until occlusion of the MCA. MCAo was confirmed after a reduction in cerebral blood flow (CBF) of at least 80% using laser-Doppler (Moor Instruments, UK). The filament was withdrawn after 30 min to establish reperfusion. Throughout surgery, core body temperature was maintained at 37 ± 0.5℃ using a homeothermic blanket and monitored using a rectal probe (Harvard Apparatus, UK). Sham-operated animals were prepared where the filament was advanced along the internal carotid artery and immediately withdrawn. After surgery, all mice were given subcutaneous saline and mashed chow diet. In total, 10% of mice died during or after the surgical procedure for MCAo and 3% were excluded due to a subarachnoid haemorrhage. No deaths or complications occurred in sham-operated animals. Surgery was not performed in chronic high-fat fed mice as we have reported previously increased ischaemic damage in these mice.^7^ Mice were allowed to recovery for 24 h following MCAo.

### Tissue preparation

All mice were terminally anesthetised with isoflurane (3–4% in 30% O_2_ and 70% N_2_O) and cardiac blood was taken using 3.8% sodium citrate as an anticoagulant, and plasma obtained after centrifugation (1200 *g* for 30 min at 4℃) was stored at −80℃ until assay. Animals were then perfused transcardially with 0.9% saline and the liver, epididymal adipose tissue and brain collected. In naïve mice, half the liver and adipose tissue samples were fixed overnight in 4% paraformaldehyde (PFA) for subsequent histology, and the other half snap frozen in isopentane and stored at −80℃ for subsequent homogenisation. For MCAo and sham surgery, the brain was snap frozen in isopentane and coronal sections (20 µm) were cut on a cryostat (Leica, Germany). At the end of each series of thin cut sections, a thick cut section (150 µm) was taken. For each thick cut section, the hemispheres were separated, and the ipsilateral and contralateral hemispheres pooled for each animal for subsequent homogenisation. The 20-µm section was used for analysis of ischaemic damage (see below).

### Ischaemic damage

Coronal brain sections (20 µm) were stained with haematoxylin and eosin (H&E). Ischaemic damage (corrected for oedema) was then measured and calculated as infarct volume by measuring the areas of cell death at eight anatomically defined coronal levels as previously described.^31^ Briefly, areas of cell death were confirmed by light microscopy, recorded on printed brain maps, which were then digitized.

### Brain, liver and adipose cytokine/chemokine analysis

Ipsilateral (ischaemic) brain samples and liver and adipose tissue were homogenised in buffer (50 mM Tris–HCl, 5 mM CaCl_2_, 150 mM NaCl and 0.02% NaN_3_), consisting of 1% Triton-X and a protease inhibitor cocktail and centrifuged at 10,000 × *g* for 30 min at 4℃. The supernatant was then collected and stored at −20℃ until analysis. Monocyte chemoattractant protein-1 (MCP-1/CCL2), macrophage inflammatory protein-1α (MIP-1α/CCL3), granulocyte colony-stimulating factor (G-CSF), chemokine (C-X-C motif) ligand 1 (CXCL1/KC), interleukin-6 (IL-6), tumor necrosis factor alpha (TNFα), intracellular adhesion molecules (ICAM) and IL-12 were analysed by enzyme-linked immunosorbent assay (ELISA; R&D Systems, UK) according to the manufacturer’s instructions. Cytokine/chemokine concentrations were determined by reference to the relevant standard curves. Protein concentration was assessed by a bicinchoninic protein assay (BCA; Pierce Biotechnology, USA), and results expressed as pg/mg or ng/mg protein.

### Immunoblotting

Antibodies for immunoblotting were as follows: anti-GLUT1 (1:1000, Merck Millipore), anti-β-actin (1:5000, Sigma). Homogenised ipsilateral (ischaemic, I) and contralateral (non-ischaemic, NI) brain samples (25 µg of protein for each sample) were separated by SDS page, and proteins transferred to a polyvinylidene fluoride membrane. After being blocked (5% milk and 0.1% TWEEN-20 in PB-Saline (PBS-T)), membrane was incubated at 4℃ overnight in primary antibodies diluted in 1% BSA in PBS-T. Membranes were then incubated with HRP-conjugated secondary antibodies (1:500, Wako Chemicals) in 5% milk in PBS-T and blots developed using an Enhanced Chemiluminescent Detection Kit (GE Healthcare). Digital images of protein bands were acquired (ImageQuant 350, GE Healthcare, UK) and semi-quantitative analysis of protein content performed by densitometry using ImageQuant TL software (GE Healthcare, UK), with β-actin used as a loading control.

### Data and statistical analyses

All data are presented as mean ± standard error of the mean (S.E.M). Sample sizes were determined by power calculation (α = 0.05, β = 0.2) of our previous data. All ex vivo quantification was done blinded to diet and treatment (MCAo or sham). All statistical tests were performed using SPSS (IBM SPSS Statistics), with *P* < 0.05 considered as significant. For parameters in naïve animals, data were analysed using a one-way ANOVA with Bonferroni post h*oc* test. Ischaemic damage was analysed by a Mann–Whitney U test. Other comparisons were made by two-way analysis of variance with Bonferroni post hoc test with diet and treatment (sham versus stroke/MCAo, or non-ischaemic versus ischaemic hemisphere) as fixed factors. All reporting of animal experiments complied with the ARRIVE guidelines (Animal Research: Reporting in In Vivo Experiments).

## Results

### An acute high-fat diet had no effect on body weight, metabolic and inflammatory parameters or adipose tissue and liver pathology

**Figure 1.**
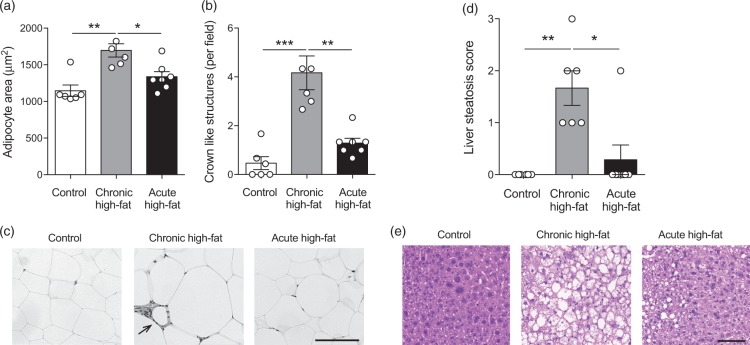
An acute high-fat diet has no effect on adipose tissue and liver pathology. Mice (eight-week-old) were fed a control or a high-fat diet (chronic high-fat) for 7.5 months, or a control diet for 7.5 months followed by a high-fat diet for three days (acute high-fat). Adipose tissue cell area (a) and the number of crown-like clusters (b) were then assessed in epididymal adipose tissue, and liver steatosis (d) scored. Images are representative sections of adipose tissue (c) or liver (e) stained with haemotoxylin and eosin. Arrow in (c) represents a crown-like cluster around an adipocyte. Scale bars = 100 µm. Data are mean ± SEM, *n* = 5–7/group. N.B. One naïve chronic high-fat fed mouse had damaged adipose tissue due to sample preparation so for these analyses *n* = 5 for this group. *P < 0.05, **P < 0.01, ***P < 0.001; One-way ANOVA with Bonferroni post hoc analysis.

In naïve mice, a chronic high-fat diet (7.5 months) led to a significant (*P* < 0.001) increase (60%) in body weight (Table 1) when compared to control-fed mice. This increase in body weight in chronic obese mice was accompanied by a significant increase (∼40%) in adipocyte cell area (*P* < 0.01) and the number of crown like structures (6-fold increase; *P* < 0.001) that are representative of macrophages clusters around dead adipocytes (Figure 1(a) to (c)).^30^ Mice fed a chronic high-fat diet also developed significant steatosis in the liver (Figure 1(d) and (e)). In contrast, no change in body weight (Table 1) and adipocyte size, the appearance of adipocyte crown-like clusters or liver steatosis was detected in mice fed a high-fat diet for three days (acute high-fat, Figure 1(a) to (e)).

**Table 1.** Effect of an acute versus chronic high-fat diet on physiological, metabolic and inflammatory parameters.

	Control	Chronic high-fat	Acute high-fat
Body weight (g)	35.0 ± 1.9	56.0 ± 1.7***	37.1 ± 2.0
Systolic B.P (mmHg)	112.4 ± 0.9	115.0 ± 6.0	119.0 ± 5.0
Heart rate (bpm)	638.0 ± 19.0	658.0 ± 19.0	672.0 ± 29.0
Plasma			
Triglycerides (mM)	1.7 ± 0.2	2.2 ± 0.1*	1.7 ± 0.1
Free fatty acids (µM)	635.0 ± 120.0	735.0 ± 51.0	784.0 ± 69.0
Glycerol (mM)	2.1 ± 0.1	2.5 ± 0.1*	2.3 ± 0.1
Adipose tissue			
IL-6 (pg/mg)	54.2 ± 16.2	15.8 ± 3.2*	39.0 ± 9.7
TNFα (pg/mg)	132.5 ± 64.6	147.9 ± 39.3	87.0 ± 22.1
ICAM-1 (ng/mg)	3751 ± 1706	4148 ± 1036	2565 ± 587
Liver			
IL-6 (ng/mg)	2.7 ± 0.2	2.5 ± 0.3	2.6 ± 0.2
TNFα (ng/mg)	2.1 ± 0.1	3.7 ± 0.6*	2.1 ± 0.4

Note: Mice were kept on a control or a high-fat diet (chronic high-fat) for 7.5 months or a control diet for 7.5 months followed by a high-fat diet for three days (acute high-fat). Body weight, systolic blood pressure (B.P.) and heart rate were then assessed in conscious mice. After mice were culled, blood was taken and plasma triglycerides, free fatty acids and glycerol measured. Adipose tissue and liver homogenates were also prepared for analysis of interleukin-6 (IL-6), tumor necrosis factor alpha (TNFα) and intracellular adhesion molecule-1 (ICAM) by ELISA, and data were normalized to milligram of total protein (measured by BCA assay). Data are mean ± SEM, *n* = 6–7/group. *P < 0.05, ***P < 0.001, versus control fed mice. One-way ANOVA with Bonferroni post hoc analysis.

As a chronic high-fat diet in mice is a known driver of metabolic syndrome, serum metabolite concentrations, blood pressure and inflammatory markers were quantified (Table 1). A chronic high-fat diet caused plasma levels of triglycerides and glycerol to significantly (*P* < 0.05) increase (29% and 19%) when compared to control fed mice (Table 1). However, an acute high-fat diet did not affect serum triglycerides or glycerol. FFAs levels were not affected by diet in any group and there was no change in systolic blood pressure or heart rate after a chronic or acute high-fat diet. An acute high-fat diet also had no effect on the level of inflammatory markers IL-6, TNFα or ICAM in adipose tissue or IL-6 and TNFα in the liver, but an increase (76%) in liver TNFα and a decrease (71%) in IL-6 in adipose tissue was observed in chronic high-fat fed mice (Table 1).

### An acute high-fat diet increased plasma insulin and impaired glucose homeostasis

**Figure 2.**
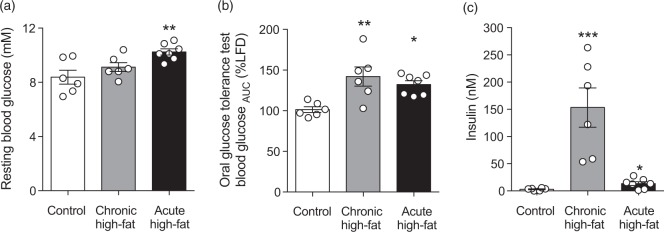
An acute high-fat diet increases plasma insulin and impairs glucose homeostasis. Mice (eight-week-old) were fed a control or a high-fat (chronic high-fat) diet for 7.5 months, or a control diet for 7.5 months followed by a high-fat diet for three days (acute high-fat). (a) Basal fasted blood glucose levels were assessed and (b) a glucose tolerance test performed after oral administration of 2 mg/kg glucose and blood glucose measured at 15, 30, 60 and 120 min. Area under the curve was analysed and is expressed as % control mice. (c) Basal plasma insulin concentrations. Data are mean ± SEM, *n* = 6–7/group. *P < 0.05, **P < 0.01, ***P < 0.001; One-way ANOVA with Bonferroni post hoc analysis.

Resting blood glucose was significantly increased (23%; *P* < 0.01) in naïve mice fed an acute high-fat diet for three days (Figure 2(a)). The area under the curve after an oral glucose tolerance challenge was also significantly increased (30%; *P* < 0.05) after acute high-fat feeding when compared to control fed mice (Figure 2(b)). In support of impaired glucose homeostasis, an increase (3.5 fold) in plasma insulin was detected after three days of high-fat diet (Figure 2(c)). A greater increase in plasma insulin (46 fold) was observed in chronically high-fat fed mice and impaired glucose homeostasis in the glucose tolerance test was observed without an increase in basal glucose (Figure 2(a) to (c)).

### An acute high-fat diet increased ischaemic damage

**Figure 3.**
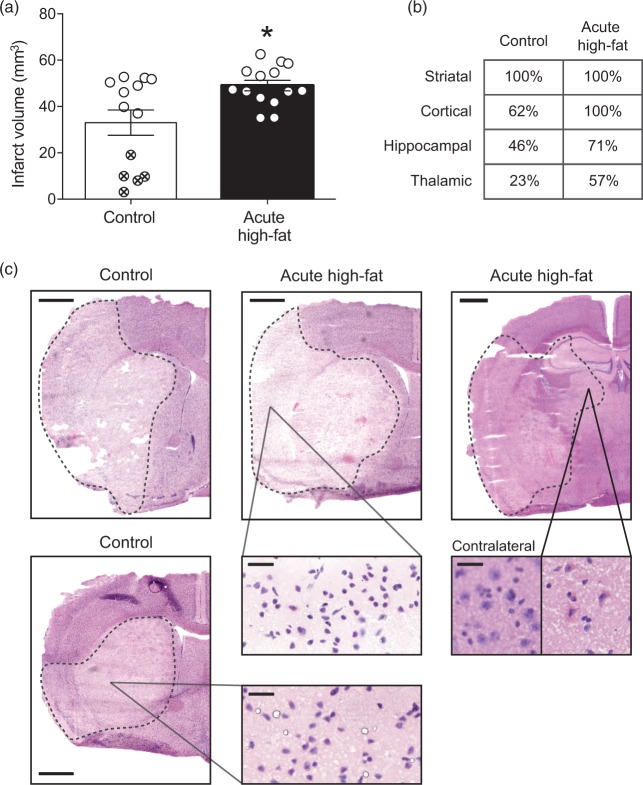
An acute high-fat diet increased ischaemic damage. Mice (eight-week-old) were fed a control diet for 7.5 months followed by a high-fat (acute high-fat) or control diet for three days. Middle cerebral artery occlusion was then performed and total infarct volume (a) and frequency of damage in defined brain regions (b) assessed at 24 h after reperfusion. Mice with only striatal damage are indicated with a crossed circle. Images are representative sections stained with haemotoxylin and eosin with the ischaemic area outlined (c). Scale bar = 1 mm, and 50 μm in insets. Data are mean ± SEM, *n* = 13–14/group. *P < 0.05; Mann–Whitney Test.

There was a significant (48%; *P* < 0.05) increase in the amount of ischaemic damage in the brains of acute high-fat fed mice 24 h after MCAo (Figure 3). The striatum was extensively damaged in both groups, with the increase in damage in the acute high-fat fed mice being due to expansion of the lesion in the cortex, hippocampus and thalamus. There was no evidence of haemorrhagic transformation in the ischaemic tissue of mice fed a control or acute high-fat diet.

### GLUT-1 expression is reduced after acute high-fat feeding and in response to stroke

**Figure 4.**
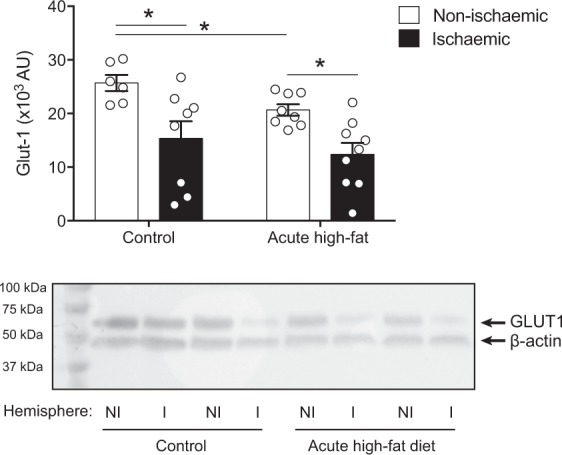
GLUT-1 expression is decreased after acute high-fat feeding and in response to stroke. Mice (eight-week-old) were fed a control diet for 7.5 months followed by a high-fat (acute high-fat) or control diet for three days. Middle cerebral artery occlusion (MCAo) to induce stroke was then performed and the ischaemic (I, ipsilateral) and non-ischaemic (NI, contralateral) hemisphere taken at 24 h. GLUT-1 expression was analysed by Western blot. Data are mean ± SEM, *n* = 6–9/group for MCAo. *P < 0.05, Two-way ANOVA with Bonferroni post hoc analysis.

In mice fed a high-fat diet for three days, the expression of the glucose transporter GLUT-1 was significantly reduced (20%; *P* < 0.05) in the contralateral non-ischaemic hemisphere compared to control-fed mice (Figure 4). The GLUT1 we detected was of the vascular 55 kDa subtype, rather than the neuronal/glial 44 kDa subtype.^32^ Stroke also led to a reduction (40%) in GLUT-1 in the ischaemic hemisphere of acute high-fat fed mice compared to the non-ischaemic hemisphere, an effect that was also observed in control fed mice (40% reduction).

### Acute high-fat diet increased free fatty acids and triglycerides after stroke

**Figure 5.**
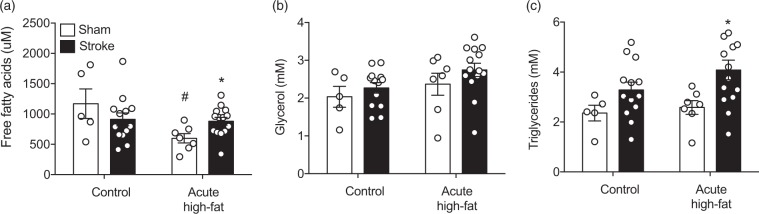
Increased plasma free fatty acids (FFA) and triglycerides after ischaemic stroke in acute high-fat fed mice. Mice (eight-week-old) were fed a control diet for 7.5 months followed by a high-fat (acute high-fat) or control diet for three days. Middle cerebral artery occlusion (MCAo) to induce stroke or sham surgery was then performed and metabolic parameters (free fatty acids (a), glycerol (b) and triglycerides (c)) assessed in the plasma at 24 h. Data are mean ± SEM, *n* = 13–14/group for MCAo and *n* = 5–7 for sham. *P < 0.05 versus sham on same diet, #*P* < 0.05 versus sham mice on control diet; Two-way ANOVA with Bonferroni post hoc analysis.

In acute high-fat fed mice, there was a significant increase (*P* < 0.05) in plasma FFA (47%) and triglycerides (58%) 24 h after MCAo compared to sham surgery, while plasma glycerol remained unchanged (Figure 5). No changes in any metabolic parameters were detected in control-fed mice 24 h after MCAo, or after sham surgery apart from a significant decrease (∼50%; *P* < 0.05) in FFA in acute high-fat fed mice.

### An acute high-fat diet had no effect on the brain cytokine/chemokine response to MCAo

**Figure 6.**
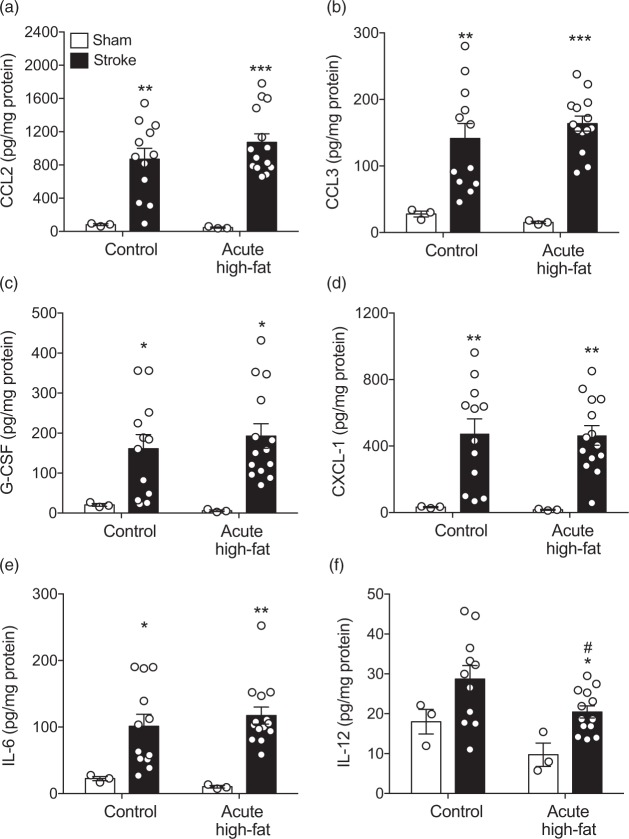
Effect of an acute high-fat diet on expression of brain cytokines and chemokines after ischaemic stroke. Mice (eight-week-old) were fed a control diet for 7.5 months followed by a high-fat (acute high-fat) or control diet for three days. Middle cerebral artery occlusion (MCAo) to induce stroke or sham surgery was then performed and brains taken at 24 h. Cytokines and chemokines were analysed in the ipsilateral hemisphere by ELISA and data corrected for mg protein. (a) Monocyte chemoattractant protein-1 (MCP-1/CCL2), (b) macrophage inflammatory protein-1α (MIP-1α/CCL3), (c) granulocyte colony-stimulating factor (G-CSF), (d) chemokine (C-X-C motif) ligand 1 (CXCL1/KC), (e) interleukin-6 (IL-6), and (f) interleukin-12 (IL-12). Data are mean ± SEM, *n* = 12–14/group for MCAo and *n* = 3 for sham. *P < 0.05, **P < 0.01, ***P < 0.001 versus sham on same diet, #*P* < 0.05 versus stroke mice on control diet; Two-way ANOVA with Bonferroni post hoc analysis.

Twenty-four hours after MCAo, there was a significant increase in CCL2, CCL3, G-CSF, CXCL-1, IL-6 in the ischaemic hemisphere of brains from mice on a control or acute high-fat diet compared to sham surgery, with no difference in the extent of the increase between the diet groups (Figure 6). IL-12 significantly increased in response to MCAo in acute high-fat fed mice only. No differences in chemokine or cytokine levels were detected after sham surgery.

In order to better compare ischaemia-induced cytokine production between diets, cytokine concentrations in brain homogenates were normalised to the ischaemic damage in each animal. This aimed to remove variations in infarct volume as a confounding factor as a greater infarct often leads to greater cytokine production. After brain homogenates were normalised to ischaemic damage, three days high-fat diet led to significantly less induction of cytokines and chemokines compared to control fed mice (Control vs. three days high-fat diet, pg/mg protein/mm^3^, CCL2, 32.8 ± 4.4 vs. 20.2 ± 1.5; CCL3, 5.3 ± 0.8 vs. 3.3 ± 0.3; G-CSF, 4.7 ± 0.4 vs. 3.3 ± 0.5; CXCL-1, 15.3 ± 1.4 vs. 9.9 ± 1.2; IL-6, 3.5 ± 0.4 vs. 2.3 ± 0.2; IL-12, 0.7 ± 0.1 vs. 0.4 ± 0.04; *P* < 0.05 for all apart from *P* < 0.01 for CCL2).

## Discussion

The present study demonstrates for the first time that consumption of a high-fat diet for only three days increased ischaemic damage after experimental stroke in mice. Acute high-fat feeding also led to a disruption in glucose homeostasis, but no change in body weight or metabolic and inflammatory status.

Several studies have demonstrated that a chronic high-fat diet leads to obesity and worse outcome after stroke in rodents.^8^ These detrimental effects of high-fat feeding in mice are apparent after four months but not at two or three months of diet.^7^ The worse outcome in obese rodents is characterized by increased ischaemic damage, cerebral oedema and haemorrhagic transformation.^8^ In the present study, acute high-fat feeding increased infarct volume, though there was no increased incidence of haemorrhage. However, 24 h post-stroke may be too soon to observe haemorrhagic transformation, and oedema may not have reached its peak severity.

Obesity as a result of prolonged high-fat feeding is often associated with liver steatosis, hyperlipidemia, low-grade inflammation and hypertension, conditions associated with the metabolic syndrome that can lead to increased risk and impact on outcome for stroke.^33–35^ Here in naïve mice, a chronic high-fat diet increased body weight and adiposity, induced liver steatosis and inflammation, and altered fat metabolism, changes not seen in mice fed an acute high-fat diet. An acute high-fat diet also had no effect on blood pressure or production of inflammatory markers from the adipose tissue or liver. Thus, it is unlikely that changes in these systems account for the negative effect of an acute high-fat diet seen here.

Obesity usually results in insulin resistance and/or type II diabetes in patients and experimental animals. Here chronic high-fat feeding in naïve mice impaired glucose tolerance and caused hyperinsulinemia. However, measures of glucose homeostasis were also perturbed after three days high-fat diet, characterised by raised blood glucose and insulin, and glucose intolerance. Impaired glucose regulation after high-fat feeding for short periods (three to four days) has been reported previously in both naïve mice,^21,23,24,36^ and also in humans fed a hypercalorific or a high-fat diet.^22,37^ Impaired glucose tolerance (or hyperglycemia) is also present in one-third of patients with a transient ischaemic attack (TIA) or ischemic stroke, and is associated with worse outcome or a two-fold risk of recurrent stroke.^38–43^ Diabetes, which is also associated with altered glucose homeostasis, increases stroke risk and mortality,^44^ and diabetic animals models have worse outcome after experimental stroke.^45^ This is likely because hyperglycaemia in rodent stroke has well-established detrimental effects on cerebral blood flow and autoregulation.^46,47^ For example, acute hyperglycemia at the time of MCAo in rodents accelerates infarct growth and increases ischaemic volume.^48^ Therefore, the hyperglycaemia resulting from a short-term high-fat diet may be leading to changes in cerebral blood flow after stroke, including reduced penumbral perfusion and an increase in the ischaemic territory.

The deprivation of blood supply during ischaemic stroke leads to a deficiency of glucose (and oxygen) in the brain, even when plasma glucose is high as seen in diabetes. The ischaemic and hypoperfused brain tissue increases its energy needs and studies have shown increased glucose uptake into the ischaemia core and penumbra after experimental stroke in rats.^49^ The glucose transporter GLUT-1 that is expressed on cerebrovascular endothelial cells mediates uptake of glucose and vitamin C into the brain.^50^ Here expression of GLUT-1 was decreased after three days of high-fat diet in naïve mice brains and stroke led to a further reduction. As GLUT-1 is one of the major brain glucose transporters, and increasing its expression is proposed to have neuroprotective potential,^51^ lower levels might reduce glucose availability in the brain and thus compromise ischaemic tissue leading to enhanced cell death especially in ‘at risk’ penumbral regions. Reduced vitamin C transport into the brain may also contribute to enhanced damage, as vitamin C is protective in cerebral ischaemia.^52^ In support, mice fed a high-fat diet for three days had impaired glucose uptake into the brain that correlated with lower levels of GLUT-1 expression possibly due to an increase in saturated free fatty acids.^53^ Furthermore, lower levels of GLUT-1 expression and a change in polarity are seen in brain endothelial cells of the spontaneously hypertensive stroke prone rat, which exhibits worse outcome after stroke.^54,55^ Alternatively, the lower levels of GLUT-1 after stroke could be a consequence of ischaemic damage and be related to the extent of injury.

Acute high-fat feeding led to a different metabolic response after stroke, compared to mice fed a control diet, with increases in free fatty acids and triglycerides detected in the plasma at 24 h. Changes in lipid metabolism have been seen in obese mice after stroke, in particular an increase in plasma free fatty acids.^56^ Increases in free fatty acids in high-fat fed mice are also observed in response to injury in other organs including the liver,^57^ and fatty acids could potentially drive pathology, especially when concentrations are inappropriately high.^58^ However, these changes in lipid production could indicate a shift in metabolism and reflect weight loss observed after stroke

Stroke is accompanied by an inflammatory response characterised by induction of pro-inflammatory cytokines and chemokines in the blood, peripheral organs and brain,^7,59,60^ which is thought to drive pathology after stroke. Here we found a robust increase in several cytokines and chemokines in the ischaemic brain 24 h after induction of stroke. Despite greater ischaemic damage after acute high-fat feeding, there was no overall difference in the level of inflammatory markers in the brain. However, when the level of inflammatory markers was normalised to infarct volume, significantly less expression was observed in the ischaemic brain of acute high-fat fed mice compared to controls. If a linear relationship exists between damage and inflammation then these data suggest that a blunted brain inflammatory response occurs after stroke in mice fed a high-fat diet for three days. A dampened inflammatory response has also been observed after stroke in mice fed a high-fat ‘diabetic’ diet.^17^ The findings in the present study are in contrast to chronic high-fat feeding and diet induced-obesity where a greater increase in brain chemokine expression is observed in the brain 24 h after stroke.^7^ Changes in the brain inflammatory response are therefore unlikely to account for increased ischaemic damage seen here after an acute high-fat diet, but could impact on recovery and repair at later time points. Furthermore, the mechanisms underlying the effect of the acute high-fat diet are likely different than those in more chronically fed mice.

In summary, we have observed that consumption of a high-fat diet for only three days was associated with changes in glucose homeostasis and an increase in ischaemic damage after stroke. A three-day high-fat diet also exacerbates ethanol-induced liver injury and inflammation^57^ suggesting that short-term high-fat feeding has detrimental effects on multiple organs. These data could therefore have implications for patients who experience a stroke after eating high-fat foods for a short period.
